# Changing Artificial Playback Speed and Real Movement Velocity Do Not Differentially Influence the Excitability of Primary Motor Cortex during Observation of a Repetitive Finger Movement

**DOI:** 10.3389/fnhum.2017.00546

**Published:** 2017-11-13

**Authors:** Takefumi Moriuchi, Daiki Matsuda, Jirou Nakamura, Takashi Matsuo, Akira Nakashima, Wataru Mitsunaga, Takashi Hasegawa, Yuta Ikio, Masahiko Koyanagi, Toshio Higashi

**Affiliations:** ^1^Unit of Rehabilitation Sciences, Nagasaki University Graduate School of Biomedical Sciences, Nagasaki, Japan; ^2^Research Fellow of the Japan Society for the Promotion of Science, Tokyo, Japan; ^3^Department of Occupational Therapy, Nagasaki University Graduate School of Biomedical Sciences Health Sciences, Nagasaki, Japan

**Keywords:** action observation, primary motor cortex, motor-evoked potentials, transcranial magnetic stimulation, mirror neuron system, video speed, movement velocity

## Abstract

Action observation studies have investigated whether changing the speed of the observed movement affects the action observation network. There are two types of speed-changing conditions; one involves “changes in actual movement velocity,” and the other is “manipulation of video speed.” Previous studies have investigated the effects of these conditions separately, but to date, no study has directly investigated the differences between the effects of these conditions. In the “movement velocity condition,” increased velocity is associated with increased muscle activity; however, this change of muscle activities is not shown in the “video speed condition.” Therefore, a difference in the results obtained under these conditions could be considered to reflect a difference in muscle activity of actor in the video. The aim of the present study was to investigate the effects of different speed-changing conditions and spontaneous movement tempo (SMT) on the excitability of primary motor cortex (M1) during action observation, as assessed by motor-evoked potentials (MEPs) amplitudes induced by transcranial magnetic stimulation (TMS). A total of 29 healthy subjects observed a video clip of a repetitive index or little finger abduction movement under seven different speed conditions. The video clip in the movement velocity condition showed repetitive finger abduction movements made in time with an auditory metronome, at frequencies of 0.5, 1, 2, and 3 Hz. In the video speed condition, playback of the 1-Hz movement velocity condition video clip was modified to show movement frequencies of 0.5, 2, or 3 Hz (Hz-Fake). TMS was applied at the time of maximal abduction and MEPs were recorded from two right-hand muscles. There were no differences in M1 excitability between the movement velocity and video speed conditions. Moreover, M1 excitability did not vary across the speed conditions for either presentation condition. Our findings suggest that changing playback speed and actual differences in movement velocity do not differentially influence M1 excitability during observation of a simple action task, such as repetitive finger movement, and that it is not affected by SMT. In simple and meaningless observational task, people might not be able to recognize the difference in muscle activity of actor in the video.

## Introduction

In recent years, several studies have described the beneficial effects of action observation interventions on motor performance. These studies have included not only healthy subjects (Stefan et al., 2005), but also patients with stroke (Ertelt et al., 2007, 2012; Bang et al., 2013), Parkinson’s disease (Pelosin et al., 2010, 2013; Agosta et al., 2017), and orthopedic disorders (Bellelli et al., 2010). In this context, the mirror neuron system (i.e., the action observation network [AON]), which is activated when an individual performs actions (e.g., goal-oriented tasks like grasping a cup) and meaningless tasks (e.g., finger abduction movement), as well as while observing other’s actions, has been implicated in the positive effects of action observation in clinical interventions (Fadiga and Fogassi, 1995; Grafton et al., 1996; Buccino et al., 2001; Rizzolatti and Craighero, 2004; Urgesi et al., 2006; Iacoboni and Mazziotta, 2007; Gatti et al., 2016). A previous longitudinal study using functional magnetic resonance imaging (fMRI) has investigated the relationship between motor function in a paralyzed upper limb after stroke and neural activation during action observation. The study found that increased activation of the cerebellum and the premotor area, representing components of the AON, correlated with improved motor function in the paralyzed limb (Brunner et al., 2014). Another study showed that “action observation therapy,” in which patients observed and subsequently mimicked video sequences depicting activities of daily living, had a significant positive effect on motor function, as compared to a control condition. Additionally, when the effects of action observation on brain activation was investigated by f-MRI, the bilateral ventral premotor cortex (PMv) and the inferior parietal lobe (IPL), which are components of the AON, showed significantly increased activation during manipulation of an object with the affected hand (Ertelt et al., 2007). Accordingly, activation of the AON during action observation has been considered to be important for improved motor function after clinical intervention (Small et al., 2012). For this reason, we believe that investigating the activation of AON during action observation would be important to establish an effective clinical intervention involving action observation.

Transcranial magnetic stimulation (TMS) has been used in previous action observation studies to show that the excitability of the primary motor cortex (M1), related to the AON, is enhanced in a manner that corresponds with the muscle(s) involved in the observed movement, and is therefore muscle-specific (Fadiga and Fogassi, 1995; Urgesi et al., 2006; Catmur et al., 2007; Catmur and Mars, 2011). This phenomenon can be explained by the hypothesis that the PMv, an important node in the AON with strong connections to M1, influences M1 activity during action observation (Fadiga and Fogassi, 1995; Avenanti et al., 2007; Koch et al., 2010; Bunday et al., 2016). We previously investigated how the video speed of observed actions affects M1 excitability, as assessed by motor-evoked potentials (MEPs) amplitudes, induced by TMS. We found that M1 excitability was significantly different for different video speeds only in a rapid movement task, but it was difficult to recognize the elements of movement (i.e., obtain kinematic information) using naked eye observation. M1 excitability was greater when subjects observed an action played at slower speed than normal or at fast speed. Conversely, M1 excitability was not influenced by manipulating the video speed during the observation of slow movements (Moriuchi et al., 2017). Previous fMRI studies have reported that the PMv and IPL constitute the parts of the AON that are particularly involved in evaluating motor-related components of observed actions, and that AON activation is dynamically modulated, depending on whether the element of movement can be recognized (Ogawa and Inui, 2012). On this premise, we hypothesized that adjusting the replay speed of a video clip of a rapid movement would make it easier to recognize the element of movement and thus to produce AON activation, particularly in the PMv and IPL, and would consequently thereby affect M1 excitability.

When we reviewed the literature on studies that addressed the relationship between “action observation” and “speed/velocity,” we identified two categories of literature: studies in which the apparent movement velocity was altered by manipulating the video speed (i.e., the video speed condition), and studies in which the real movement velocity (i.e., the movement velocity condition) was varied. In studies that manipulated the video speed, there were no reported differences in M1 excitability during the observation of an arm crank exercise played at various speeds (Wrightson et al., 2016). Alternatively, in studies that varied the actual velocity of the movement, M1 excitability was significantly correlated with the velocity of the observed movement during the observation of hand movements along curvilinear trajectories in the air (Agosta et al., 2016). Although some studies have investigated the effects of the “video speed condition” or the “movement velocity condition,” respectively, no studies have directly compared M1 excitability under video speed manipulation and movement velocity manipulation conditions for the same movement task.

A conceivable difference between the effects of the “video speed condition” and the “movement velocity condition” could involve muscle activation. In studies in which the actual movement velocity was varied, increased velocity was associated with increased burst amplitudes and areas on electromyography (EMG) (Mustard and Lee, 1987). In contrast, manipulating video speed did not produce differences in muscle activation. In the context of this example, we hypothesized that M1 excitability would be affected by muscle activity that occurred in response to the observed movement velocity during action observation.

On the other hand, some studies have considered the spontaneous movement tempo (SMT) when assessing the relationship between movement “speed/velocity” and M1 excitability during action observation. When healthy humans are asked to perform finger tapping or walk freely, most individuals use a frequency of around 2 Hz (MacDougall and Moore, 2005; Bove et al., 2009). Thus, healthy humans have a common SMT when executing the internally generated voluntary movement. The SMT influences M1 excitability during observation of the finger opposition at various movement velocity conditions (1, 2, and 3 Hz), and the highest M1 excitability is shown during observation of 2-Hz finger opposition (Avanzino et al., 2015; Lagravinese et al., 2017). If M1 excitability during action observation is affected by SMT, the highest M1 excitability would be shown at 2 Hz not only for the actual movement velocity manipulation, but also for the video speed manipulation condition.

The aim of the present study was to make a direct comparison of how changing the video speed of an observed action or changing the velocity of an actual observed action affects the excitability of M1 during action observation, as assessed by the amplitude of MEPs. Moreover, we clarified whether the effect on M1 excitability during action observation is due to changing muscle activity or due to the influence of the SMT. To this end, we here adopted repetitive finger abduction movement as an observational task. This task was chosen as it was (1) easy to control speed adjustment between the “video speed condition” and the “movement velocity condition,” (2) possible to assess changes in muscle activity due to variation in the actual movement velocity, and (3) a rhythmical repetitive movement task that can reflect the effect of SMT. The present study consisted of two experiments. In Experiment 1, we investigated whether EMG activity increased as the velocity of movement increased in the repetitive task subsequently used in Experiment 2. In Experiment 2, based on the results of Experiment 1, we investigated the influence of video speed versus actual movement velocity on M1 excitability, by assessing the amplitudes of TMS-induced MEPs while observing the repetitive finger-movement task.

## Experiment 1: EMG Study

A previous study of the relationship between muscle activity and movement velocity determined that faster movement velocities were associated with larger EMG burst amplitudes and areas (Mustard and Lee, 1987). The aim of Experiment 1 was to validate this relationship in the repetitive finger abduction task subsequently used in Experiment 2.

### Materials and Methods

#### Subjects

Twelve healthy volunteers (six men and six women, mean age: 25.7 ± 5.7 years) were enrolled in Experiment 1. All participants provided written informed consent and all were right-handed (as indicated by self-report). The study was approved by the local ethics committee at the Nagasaki University Graduate School of Biomedical Sciences. All experimental procedures were conducted in accordance with the Declaration of Helsinki (World Medical Association, 2013).

#### Experimental Set-Up

Subjects were seated on a reclining chair and were instructed to place both hands in a pronated position on a table in front of them. To maintain hand positions, the experimenter marked the point of each finger on the pad. Then, the experimenter asked subjects to abduct the index or little finger maximally and set the plate at the point of maximal finger abduction. Therefore, the range of repetitive movement was set between 0° and the plate (i.e., the point of maximal finger abduction). We recorded EMG activity while the subject performed the repetitive abduction movement five times in response to the sound of a metronome beating at 0.5, 1, 2, or 3 Hz. The speed order was randomized by the experimenter using the RAND function in Microsoft Excel, which repeatedly performed rearrangement after sorting out non-overlapping random sampling numbers. We also shoot the finger movement from above, using a web camera (c920r, Logicool, Lausanne, Switzerland; 30 fps).

#### EMG Recording

Surface EMG was recorded on the right first dorsal interosseous (FDI) muscle during repetitive index finger movement and on the abductor digiti minimi (ADM) muscle during repetitive little finger movement, using pairs of 9-mm diameter Ag - AgCl surface cup electrodes (SDC112, GE Healthcare, Osaka, Japan). Surface EMG signals were amplified and filtered at a bandwidth of 5–3000 Hz using a digital signal processor (Neuropack Sigma MEB-5504, Nihon Kohden, Tokyo, Japan), and then transferred to a computer for off-line analysis using an A/D converter (PowerLab16/30, AD Instruments, Sydney, Australia). EMG data were inputted and the finger movement movie was synchronized in real-time using data analysis software (Lab Chart 8, AD Instruments, Sydney, Australia).

#### Data Analysis

Electromyography data were analyzed using the root mean square (RMS) value of every 100 ms. The onset of muscle activity was the time point of the first maximal abduction, and offset of muscle activity was the time point of the fifth maximal abduction, as verified by monitoring the video. The mean data were statistically analyzed using Pearson’s correlation to determine the relationship between the movement velocity and EMG activity. In all analyses, the level for statistical significance was set at *p* < 0.05. All analyses were performed using statistical analysis software (SPSS version 22.0, IBM, United States).

### Results

**FIGURE 1:**
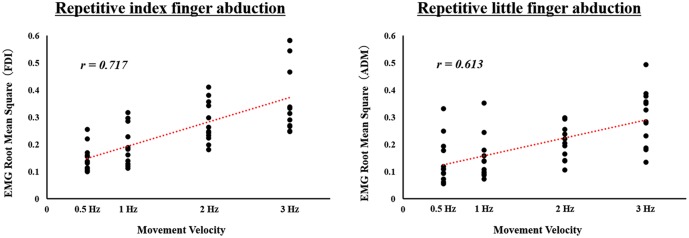
Correlation between movement velocity and electromyographic (EMG) activity during actual repetitive index or little finger abduction movement under different speed conditions in Experiment 1. Surface EMG activity calculated as the RMS for the first dorsal interosseous (FDI) muscle during repetitive index finger abduction movement, and for the abductor digiti minimi (ADM) muscle during repetitive little finger abduction movement is shown. Movement velocity is showed on the *X*-axes. EMG RMS for FDI and ADM is showed on the *Y*-axes.

We confirmed that all subjects could carry out any velocity condition tasks by assessing the video, and analyzed the data accordingly. We found significant positive correlation between movement velocity and EMG activity both in the FDI during repetitive index finger abduction (*r* = 0.717, *p* < 0.0001) and in the ADM during repetitive little finger abduction (*r* = 0.613, *p* < 0.0001). We also found that, as the velocity of movement increased, EMG activity increased (**Figure 1**).

## Experiment 2: TMS Study

Experiment 2 investigated the effects of the video speed condition versus the movement velocity condition on M1 excitability, induced by TMS, during the observation of repetitive index finger abduction movements (Task 1) and repetitive little finger abduction movements (Task 2).

### Materials and Methods

#### Subjects

Twenty nine healthy volunteers (Task 1: eight men and six women, mean age: 28.7 ± 8.7 years; Task 2: seven men and eight women, mean age: 25.8 ± 7.2 years) were enrolled in the study. One subject who participated in Task 1, and three subjects who participated in Task 2, had also participated in Experiment 1. All participants provided written informed consent and all were right-handed (as indicated by self-report). The present study was based on the global guidelines for care in the use of TMS (Rossi et al., 2009). In the first stage of recruitment, all subjects completed a questionnaire designed to exclude those with contraindications; however, no subjects reported neurological impairment or contraindications for TMS.

#### Experimental Set-Up

Subjects were seated on a reclining chair 80 cm away from a PC monitor (RDT234WX-Z, MITSUBISHI, Tokyo, Japan; 23 inches; resolution 1920 × 1080 pixels; refresh frequency 60 Hz) and were instructed to put both hands in a pronated position on a horizontal board attached to the chair’s armrests. They were instructed to keep the right forearm as relaxed and motionless as possible while paying attention to the visual stimuli presented on the PC monitor. To ensure passive observation of the video clips, the experimenter’s only instruction to the subjects was “You should stay alert while observing the hand,” prior to starting the experiment.

#### Experimental Stimuli

**FIGURE 2:**
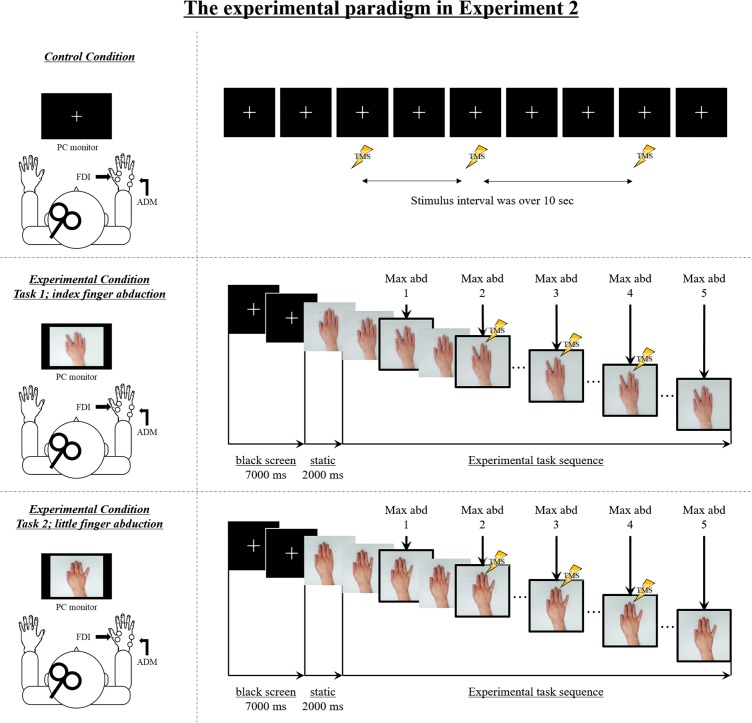
The experimental setup and sequence of still images from the video clip used in Experiment 2. Control condition: M1 excitability at rest was assessed in each subject by recording motor-evoked potentials (MEPs) from both the FDI muscle and the ADM muscle during the observation of a white cross on a black screen under controlled conditions. Transcranial magnetic stimulation (TMS) was applied at random while observing a white cross on a black screen, and the stimulus interval always exceeded 10 s. Experimental condition: The sequence of the experimental stimulus is shown. During the first 7000 ms, a white cross was presented in the center of a black screen, and during the next 2000 ms, a static hand was presented in the center of the screen. The first black screen and the static hand image were presented for a total of 9000 ms for each speed condition (0.5 Hz, 1 Hz, 2 Hz, 3 Hz, 0.5 Hz-Fake, 2 Hz-Fake, and 3 Hz-Fake). Subsequently, the repetitive finger movement sequences were displayed. The frame in the solid box is the point of maximal abduction. TMS was delivered at one of three time points (i.e., during the second, third, or fourth point of maximal abduction in the sequence of five movements) and MEPs were recorded from both FDI and ADM. As a matter of course, because the speed is different, the duration of the experimental task stimulus was also different. The stimulus lasted 10,000 ms in the 0.5 Hz/0.5 Hz-Fake conditions, 5000 ms in the 1 Hz condition, 2500 ms in the 2 Hz/2 Hz-Fake conditions, and 1667 ms in the 3 Hz/3 Hz-Fake condition, from the beginning of repetitive finger abduction movement.

To encode the experimental stimuli, we recorded video for four different speed conditions from a first-person perspective. Videos were recorded using a web camera (c920r, Logicool, Lausanne, Switzerland; 30 fps). An actor performed five repetitions of index and little finger abduction movements in time with an auditory metronome set at frequencies of 0.5, 1, 2, or 3 Hz. In the movement velocity condition, we used the original video. In the video speed condition, we changed the playback speed of the 1-Hz video from the movement velocity condition to show frequencies of 0.5, 2, and 3 Hz. We used the 1-Hz video for editing the video speed condition to demonstrate whether the highest M1 excitability would also affect the SMT (i.e., as the highest M1 excitability is shown in the 2-Hz condition) in the video speed condition, as demonstrated previously. A sequence of still images from the video clip used in the present study is shown in **Figure 2**.

#### TMS Timing

To set the trigger stimulation, the video file (.wmv file) recorded on the web camera was converted to pictures file (.jpeg file) and shown in succession to produce an animation effect. The presentation time of each frame was twice the length of the refresh interval (33.3 ms/frame) used by the PC monitor (refresh interval = 16.67 ms). The timing of the TMS trigger was established for each specific file: it occurred at the same point in the action on the video. The TMS pulse was applied at the timing of maximal abduction between the second and fourth repetitions, as per a previous action observation study that used index and little finger abduction movement tasks (Aziz-Zadeh et al., 2002; Wright et al., 2014). The file number that was set as the TMS trigger at the second, third, and fourth maximal abduction from the beginning of the sequence in each speed condition is shown in **Table 1**.

**Table 1:** Lists of the file number from the beginning of the sequence.

			Point of maximal abduction				
	Black screen	Static	1	2	3	4	5
0.5 Hz/0.5 Hz-Fake	1-210	211-271	331	391	451	511	571
1 Hz	1-210	211-271	301	331	361	391	421
2 Hz/2 Hz-Fake	1-210	211-271	286	301	316	331	346
3 Hz/3 Hz-Fake	1-210	211-271	281	291	301	311	321

*The file number that was set as the transcranial magnetic stimulation (TMS) trigger at the second, third, and fourth maximal abduction from the beginning of the sequence in each speed condition*.

Prior to the action observation task, M1 excitability at rest was assessed in each subject by recording 12 MEPs during the observation of a white cross on a black screen under controlled conditions. The TMS stimulus was applied at random, and the stimulus interval was always over 10 s (**Figure 2**). Subsequently, the experimenter instructed the subject to observe the experimental video without any additional mental effort. All subjects observed the video clips played at seven different speed conditions (four movement velocity condition [0.5, 1, 2, and 3Hz] and three video speed conditions [0.5, 2, and 3 Hz-Fake]). TMS was delivered once during each video clip, randomly at one of the three TMS pulse points. Each speed condition was viewed in 12 trials; four trials delivered the pulse during the second abduction movement, four trials during the third abduction movement, and four trials during the fourth abduction movement. The speed order was randomized by the experimenter using the RAND function in Microsoft Excel, which repeatedly performed rearrangement after sorting out non-overlapping random sampling numbers. We used a computerized pulse-generation system (LabView, National Instruments, Austin, TX, United States) to randomize the order and to ensure that the TMS trigger was always delivered at the correct time.

#### TMS and MEP Recordings

Transcranial magnetic stimulation employed a 70-mm figure-of-eight coil connected to a magnetic stimulator (Magstim 200; Magstim, Whitland, United Kingdom). At the beginning of the experiment, the optimal TMS coil position for evoking MEPs in both the right FDI and the right ADM, called the “hotspot,” was identified. TMS was delivered to the left M1 hotspot, which was marked with a pen on a swimming cap covering the subject’s scalp. The coil was placed tangentially to the scalp with its handle pointing backward and rotated approximately 45° away from the mid-sagittal line. Care was taken to maintain the same coil position relative to the scalp throughout the experiment. The same investigator administered TMS throughout the study. The resting motor threshold was defined as the lowest stimulus intensity that evoked a MEP of at least 50 mV in amplitude in the right FDI and ADM in 5 out of 10 trials. The test stimulus intensity was set to 110–130% of the resting motor threshold and elicited 0.5–1.0 mV MEPs. Throughout the experiments, subjects were instructed to avoid inadvertent movements that could raise the EMG background. For each muscle in each trial, the 20-ms period preceding the TMS trigger was checked for background EMG activity.

Surface EMG activity was recorded from the FDI and ADM using pairs of 9-mm diameter Ag - AgCl surface cup electrodes (SDC112, GE Healthcare, Osaka, Japan). Surface EMG signals were amplified and filtered at a bandwidth of 5–3000 Hz using a digital signal processor (Neuropack sigma MEB-5504, Nihon Kohden, Tokyo, Japan) and were transferred to a computer for offline analysis using an A/D converter (PowerLab16/30, AD Instruments, Sydney, Australia).

#### Data Analysis

If a background EMG signal was detected, data from both muscles in the trial were rejected. MEP amplitudes (peak-to-peak) were measured for each muscle in each trial. To investigate differences in MEP amplitudes among the experimental conditions, peak-to-peak MEP amplitude values were expressed as percentages of the mean amplitude under control conditions. The data were analyzed statistically using three-way ANOVA analysis with the factors “muscle” (FDI, ADM), “speed” (0.5 Hz, 1 Hz, 2 Hz, 3 Hz, 0.5 Hz-Fake, 2 Hz-Fake, and 3 Hz-Fake), and “speed presentation condition” (video speed condition, movement velocity condition). We employed paired *t*-tests for paired comparisons or Bonferroni *post hoc* tests for multiple comparisons. In all analyses, the level for statistical significance was set at *p* < 0.05. All analyses were performed using statistical analysis software (SPSS version 22.0, IBM, United States).

## Results

### Typical MEP Waveforms

**FIGURE 3:**
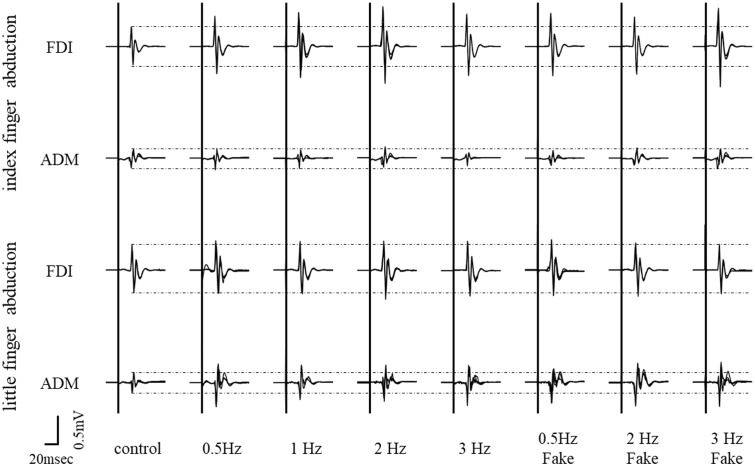
Typical superimposed waveforms of MEP amplitudes from the FDI muscle and ADM muscle in three trials under seven different speed conditions, recorded from a representative subject in Experiment 2.

Typical superimposed waveforms of MEP amplitudes of the right FDI and ADM in three trials at different speeds, recorded from a representative subject, are shown in **Figure 3**. For the muscle engaged in the movement (i.e., index finger movement – FDI, little finger movement – ADM), there was a tendency for MEP amplitudes to be higher during the observational task than during the control condition. On the other hand, there were no differences in MEP amplitudes among the different speed conditions for either muscle.

### Mean MEP Amplitudes at Each Speed and Presentation Condition

**FIGURE 4:**
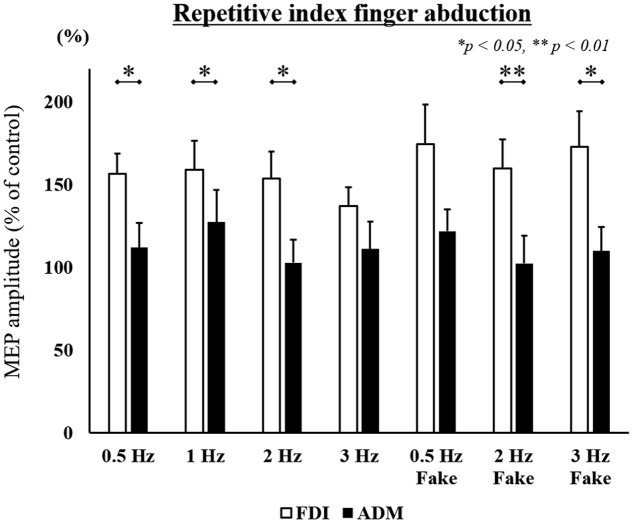
MEP amplitudes from the right FDI muscle and ADM muscle in seven different speed conditions during the repetitive index finger abduction movement task in Experiment 2. Values are expressed as percentages of the control condition amplitude (*n* = 14). The asterisk (^∗^) represents *p* < 0.05 and (^∗∗^) represents *p* < 0.01 for comparing FDI and ADM data.

**FIGURE 5:**
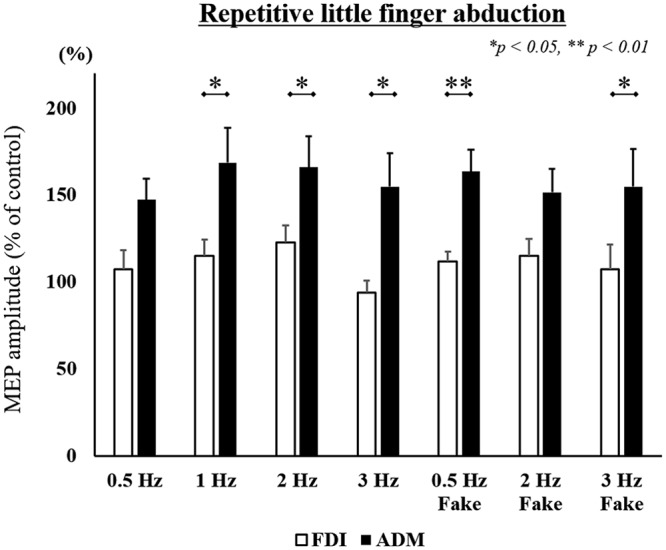
MEP amplitudes from the right FDI muscle and ADM muscle in seven different speed conditions during the repetitive little finger abduction movement task in Experiment 2. Values are expressed as percentages of the control condition amplitude (*n* = 15). The asterisk (^∗^) represents *p* < 0.05 and (^∗∗^) represents *p* < 0.01 comparing FDI and ADM data.

Mean MEP amplitudes, as a percentage of control (±standard error) induced in the right FDI and ADM in response to a single-pulse TMS are shown in **Figures 4** and **5**. A three-way ANOVA revealed a significant main effect of “muscle” [repetitive index finger movement in Task 1: *F*_(1,182)_ = 24.536, *p* < 0.001; repetitive little finger movement in Task 2: *F*_(1,196)_ = 36.663, *p* < 0.001]. There were no significant main effects for “speed” or “speed presentation condition” (*speed*, repetitive index finger movement in Task 1: *F*_(3,182)_ = 0.661, *p* = 0.577; repetitive little finger movement in Task 2: *F*_(3,196)_ = 0.617, *p* = 0.605; *speed presentation condition*, repetitive index finger movement in Task 1: *F*_(1,182)_ = 1.287, *p* = 0.258; repetitive little finger movement in Task 2: *F*_(1,196)_ = 0.063, *p* = 0.802), or any interactions.

To compare differences in MEP amplitudes in the FDI and ADM among different speed conditions, *post hoc* pairwise comparisons were performed using paired *t*-tests and revealed that MEP amplitudes were significantly higher in the FDI than in the ADM during observation of the repetitive index finger movement (Task 1) at the 0.5 Hz, 1 Hz, 2 Hz, 2 Hz-Fake, and 3 Hz-Fake conditions. Moreover, MEP amplitudes tended to be higher in the FDI than in the ADM at 0.5 Hz-Fake (*p* = 0.060). Conversely, MEP amplitudes were higher in the ADM than in the FDI during observation of the repetitive little finger movement (Task 2) at the 1 Hz, 2 Hz, 3 Hz, 0.5 Hz-Fake, and 3 Hz-Fake. Furthermore, MEP amplitudes tended to be higher in the ADM than in the FDI at 0.5 Hz (*p* = 0.052) and 2 Hz-Fake (*p* = 0.056) (**Table 2**). Therefore, changes in M1 excitability during action observation were muscle-specific, which was consistent with the findings of previous studies (Fadiga and Fogassi, 1995; Urgesi et al., 2006; Catmur et al., 2007; Catmur and Mars, 2011).

**Table 2:** Lists of motor-evoked potential (MEP) amplitudes in Experiment 2.

	Task 1: Index finger movement			Task 2: Little finger movement		
	FDI	ADM	*p*-value	FDI	ADM	*p*-value
0.5 Hz	155.6 ± 12.0	111.9 ± 15.2	<0.05	107.1 ± 11.1	147.1 ± 12.2	=0.052
1 Hz	158.9 ± 17.4	127.3 ± 19.3	<0.05	114.8 ± 9.5	165.8 ± 20.1	<0.05
2 Hz	153.5 ± 16.3	102.6 ± 14.1	<0.05	122.5 ± 9.9	165.9 ± 17.7	<0.05
3 Hz	137.0 ± 11.5	111.0 ± 16.5	=0.156	93.9 ± 6.6	154.8 ± 19.2	<0.05
0.5 Hz-Fake	174.4 ± 23.9	121.9 ± 13.0	=0.060	111.9 ± 5.6	163.4 ± 12.6	<0.01
2 Hz-Fake	159.8 ± 17.5	102.3 ± 16.9	<0.01	114.9 ± 9.9	151.5 ± 13.6	=0.056
3 Hz-Fake	172.7 ± 21.6	109.8 ± 14.6	<0.05	107.4 ± 13.9	154.8 ± 21.7	<0.05

*FDI, first dorsal interosseous, ADM, abductor digiti minimi (mean ± standard error)*.

## Discussion

In the present study, the main findings were that (1) as the velocity of repetitive finger movement increased, the EMG activity increased, (2) M1 excitability was increased in the muscle area involved in the execution of the observed actions (i.e., muscle-specific), and (3) that the effect of M1 excitability during action observation did not differ between the “video speed condition” and the “movement velocity condition” with respect to a repetitive finger-movement task.

### Relationship between Movement Velocity and EMG Activity

The present study revealed that, as the velocity of repetitive finger movement increased, the EMG activity in the muscle involved in the movement increased. Similar results had previously been shown not only in the intrinsic hand muscle, but also in the shoulder muscle and a few other muscles (Arndt, 1987; Mustard and Lee, 1987; Laursen et al., 1998). As we were able to verify this relationship, the repetitive finger-movement task was adopted as the observational task in Experiment 2.

### Video Speed Condition

A previous study comparing differences in the effects of manipulating the speed of a viewed task (i.e., video speed condition) revealed that M1 excitability during passive observation was only increased when observing a rapid movement on a video played back slowly, but not when it was played at normal or fast speeds (Moriuchi et al., 2014, 2017), indicating that M1 excitability was only altered when the element of movement could be easily recognized. Similarly, other previous studies suggested that the excitability of M1 during passive observation was not modulated in high-speed video playback conditions (Wrightson et al., 2016; Moriuchi et al., 2017). As per our previous study (Moriuchi et al., 2014, 2017), we hypothesized that M1 excitability during action observation would be influenced by activation of the PMv and IPL, as constituents of the AON, which are dynamically modulated depending on whether the element of movement is recognized or not. Because subjects were able to recognize movement elements easily, our findings indicate that the modulation of M1 excitability may not be detectable or present during the observation of videos of simple movement tasks, such as repetitive finger abduction, played at different playback speeds.

### Movement Velocity Condition

In previous movement velocity studies, increases in movement velocity were associated with increased EMG activity. A recent study in monkeys also revealed a linear relationship between EMG activation and M1 excitability (Townsend et al., 2006). Moreover, human neuroimaging studies using TMS, fMRI, positron-emission tomography, and electroencephalography have similarly revealed a positive relationship between movement velocity and M1 excitability during the actual performance of movement and motor imagery (Rao et al., 1996; Lutz et al., 2005; Yuan et al., 2010; Wang et al., 2017). We therefore hypothesized that M1 excitability would be modulated as a result of EMG activation in response to observed movement velocity, as suggested in previous studies. We tested this hypothesis in the present study, but did not detect any differences among the movement velocity conditions.

It has been suggested that AON activation is higher during the observation of goal-oriented or meaningful movements involving an object, than during the observation of meaningless movements that are not associated with a goal or object (Agnew et al., 2012; Gatti et al., 2016). In a simple and meaningless task, such as repetitive finger abduction, changing movement velocity in the absence of a clear objective was likely insufficient to affect activation of the AON. Thus, a future study should examine differences in EMG activity and M1 excitability among different movement velocity conditions using a goal-oriented simple finger-movement task.

However, it is notable that in previous studies, even meaningless tasks were useful for demonstrating the modulation of M1 excitability in response to observed movement velocity. In such a previous study, the observed movement involved writing a trigram in the air with both hands. This study suggested that M1 excitability was modulated by the properties of kinematics and the velocity of the observed movement during action observation (Agosta et al., 2016). It has been reported that the parietal parts of the AON respond to the kinematic characteristics(Iacoboni et al., 1999; Dahan and Reiner, 2015). Thus, even in a meaningless task, upper limb movement may represent more dynamic movement, with different types of movement velocities, than simple finger movement. Therefore, we suggested that, even for the same type of meaningless task, differences in performing the task was the result of whether subjects could recognize obvious differences in kinematic characteristics.

### Relationship between the Video Speed and Movement Velocity Conditions

The most notable difference between the video speed and movement velocity conditions was in EMG activity; when EMG activity was increased due to increased velocity in the repetitive finger abduction movement task, kinematic characteristics, such as muscle bulge, tremor, and skin tone changed. In contrast, these characteristics did not change in the video speed condition. A previous TMS study of action observation showed that M1 excitability during the observation of grasping and lifting of objects with different weights was modulated by changes in kinematic characteristics, such as upper limb or hand trajectory, muscle contraction, and skin tone (Alaerts et al., 2010). Considering this previous investigation, we assumed that M1 excitability only increased when kinematic changes were evident, independent of apparent movement speed. However, the present study did not reveal different activation patterns between the conditions.

On the other hand, it has also been proposed that the effect of SMT representing the highest M1 excitability is seen during observation of a 2-Hz finger opposition task (a rhythmical repetitive movement task) (Avanzino et al., 2015; Lagravinese et al., 2017). We hypothesized that if the highest M1 excitability is shown during a 2-Hz-Fake condition (i.e., changing video speed condition), the effect of changing speed on M1 excitability during action observation would not affect differences in EMG activity, which are due to the changing actual movement velocity but the effect of SMT. However, we could not test our hypothesis on the role of SMT, as there were no differences between the “movement velocity condition” and “video speed condition.” The present study suggested that repetitive finger abduction movement may not affect the SMT. The differences between the effect of a “movement velocity condition” and a “video speed condition,” using finger opposition movement as the experimental task, should be investigated further in future.

The findings of the present study should be interpreted with consideration that the present study (1) used a meaningless movement task and (2) that there were small differences in kinematic characteristics between the video speed and movement velocity conditions. Future studies should examine M1 excitability during the observation of a goal-oriented task or a task involving an object that results in more obvious changes in kinematic patterns as a result of changes in movement velocity, and additionally should consider how M1 excitability relates to AON activation during action observation.

### Clinical Implications

Some recent studies have shown a positive effect in terms of the clinical application of action observation in terms of changing speed. Parkinson’s disease patients demonstrate bradykinesia of finger movements as a characteristic of their condition; they were able to increase their spontaneous finger movement rate while observing a movement video demonstrating a rate that was faster than their own movement rate (Pelosin et al., 2013). Another study that examined the effect of changing the playback speed of a video of an arm crank in healthy subjects demonstrated that a faster playback video condition improved the participants’ cadence and power (Wrightson et al., 2016). Although not a study of “action observation,” but rather one of “motor imagery,” another study described the use of motor imagery at different speeds in athletes, according to the stage of motor learning (Jenny and Hall, 2009). It can be argued that there is a relationship between action observation and speed in terms of effective clinical application.

Both the conditions of “manipulating the video speed” and “changing actual movement velocity” revealed the effect of improving motor performance. However, it is still unknown which method of intervention is effective for action observation. Therefore, our study was conducted based on a neurophysiological index; however, the present study did not find any differences in the effects between these conditions. This should be investigated further in future.

## Conclusion

In the present study, we explored the influence of video speed versus actual movement velocity on M1 excitability as it relates to the AON during action observation. We did not identify any differences in M1 excitability between the video speed and movement velocity conditions; moreover, M1 excitability did not vary in association with increased video speed or movement velocity. In conclusion, we suggest that changes in the task speed during a simple single joint movement do not influence M1 excitability in relation to AON during action observation.

## Author Contributions

TMo, DM, THa, and THi conceived and designed the experiments. TMo, YI, and THi performed the experiments. TMo, AN, and THi analyzed the data. TMo, WM, MK, and JN created the experimental program. TMo drafted the manuscript. TMa and THi helped with the writing the manuscript. All authors approved the final version of the manuscript.

## Conflict of Interest Statement

The authors declare that the research was conducted in the absence of any commercial or financial relationships that could be construed as a potential conflict of interest.
